# Cortical Oxygenation during a Motor Task to Evaluate Recovery in Subacute Stroke Patients: A Study with Near-Infrared Spectroscopy

**DOI:** 10.3390/neurolint14020026

**Published:** 2022-03-23

**Authors:** Nicola Lamberti, Fabio Manfredini, Francesca Nardi, Andrea Baroni, Giovanni Piva, Anna Crepaldi, Nino Basaglia, Ilaria Casetta, Sofia Straudi

**Affiliations:** 1 Department of Neuroscience and Rehabilitation, University of Ferrara, Via Luigi Borsari 46, 44121 Ferrara, Italy; nicola.lamberti@unife.it (N.L.); francesca.n91@gmail.com (F.N.); anna.crepaldi@edu.unife.it (A.C.); ilaria.casetta@unife.it (I.C.); sofia.straudi@unife.it (S.S.); 2 Unit of Rehabilitation Medicine, University Hospital of Ferrara, Via Aldo Moro 8, 44124 Ferrara, Italy; a.baroni@ospfe.it (A.B.); n.basaglia@ospfe.it (N.B.); 3 PhD Program in Environmental Sustainability and Wellbeing, University of Ferrara, Via Paradiso 12, 44121 Ferrara, Italy; giovanni.piva@unife.it; 4 PhD Program in Biomedicine, Instituto Maimónides de Investigación Biomédica de Córdoba, 14005 Córdova, Spain; 5 Unit of Clinical Neurology, University Hospital of Ferrara, Via Aldo Moro 8, 44124 Ferrara, Italy

**Keywords:** rehabilitation, stroke, near-infrared spectroscopy, exercise therapy, exercise testing, motor task, cortical activity

## Abstract

In subacute stroke patients we studied cortical oxygenation changes by near-infrared spectroscopy (NIRS) during a motor task performed with the hemiparetic arm (15 s of reaching and grasping, 45 s of rest, repeated 6 times). Twenty-three subjects were included at baseline, compared with six healthy subjects, and restudied after 6 weeks of rehabilitation. Motor/premotor cortical changes in oxyhemoglobin detected by NIRS were quantified as the area under the curve (AUC) for the total cortex (TOT-_AUC_) and for both affected (AFF-_AUC_) and unaffected hemispheres (UN-_AUC_). The ratio between AUC and the number of task repetitions performed identified the cortical metabolic cost (CMC) or the oxygenation increase for a single movement. Fugl–Meyer assessment of the upper extremity (FMA-UE) was also performed. At baseline, both total and hemispheric CMC were significantly higher in stroke patients than in healthy subjects and inversely correlated with FMA-UE. After rehabilitation, changes in total-CMC and unaffected-CMC, but not Affected-CMC, were inversely correlated with variations in the FMA-UE score. A value > 5000 a.u. for the ratio baseline TOT-_CMC_/days since stroke was associated with not reaching the clinically important difference for FMA-UE after rehabilitation. In subacute stroke the CMC, a biomarker assessed by NIRS during a motor task with the hemiparetic arm, may describe cortical time/treatment reorganization and favor patient selection for rehabilitation.

## 1. Introduction

Stroke is one of the leading causes of disability worldwide with ischemic stroke which accounts for the majority of the events [1,2]. Oxygen availability underpins the function of the brain, which consumes 20% of the available oxygen when representing only 2% of the body mass [3]. The hemodynamic pattern also plays a role in the diagnostic phase and in the study of residual function after stroke considering that noninvasive techniques such as functional magnetic resonance imaging (fMRI) and functional near infrared spectroscopy (fNIRS) [4,5,6,7] exploit the hemodynamic perturbation in response to brain activation. As fMRI enables the study of brain activity by analyzing blood flow changes in active regions on the basis of the coupling between blood flow and neuronal activation [8], NIRS by several optic fibers similarly detects local changes in oxygenated hemoglobin in response to increased energy demand related to neural activation during motor or cognitive tasks [5,9]. When comparing these techniques in a sample of stroke patients during a motor task, a very similar cerebral cortical activation was observed [10,11]. In addition, fMRI detected a deeper activation and a higher spatial resolution than fNIRS, which conversely showed a dynamic profile of activation [5,6,10,12]. In general, the NIRS technique showed higher applicability for its use in various environments and activities, including rehabilitation settings, being portable, less sensitive to motion artifacts and usable during dynamic movements, and at relatively lower cost [13].

As a result, in recent years, several authors have used this technique to study functional reorganization after brain damage or neurorehabilitative interventions in stroke survivors [5,14,15], even if only few longitudinal studies have reported both the effects of rehabilitative programs and the cerebral hemodynamic responses assessed by NIRS [9,15]. After all, among the several different applications of NIRS, the identification of specific biomarkers or laboratory measurements informing on disease-related events underlying behavioral state and its evolution [16,17] could be of interest in stroke as in various diseases [18,19,20]. These biomarkers may objectively detect or predict adaptive responses to interventions after stroke, when hemodynamic and oxygen consumption changes occur in neurovascular units at lower perfusion or at a higher supportive involvement [16]. However, few papers have addressed the search for a biomarker useful to follow cortical metabolic engagement in stroke survivors during upper limb motor recovery, as recently reviewed [15]. These studies required hand grasping or finger/wrist extension movements [10,21,22] using a block design based on the repetition of the tasks interspersed with resting times to detect brain activation in each working period to be averaged in the phase of analysis.

We recently completed a trial involving subacute stroke patients with upper limb paresis aimed at exploring the effects of a proximal arm robot-assisted therapy plus hand functional electrical stimulation compared with intensive conventional rehabilitation [23]. In a sample of these patients, we also assessed cortical oxygenation by NIRS during a standardized motor task involving the upper limb [10]. We hypothesized that the quantification of cortical oxygenation in a dynamic phase to calculate the cortical metabolic cost (CMC) of the movement, may represent a biomarker useful to follow the recovery processes in addition to what is derived from routinary tests. The study aims to validate this NIRS biomarker by determining whether (i) its collection is feasible in clinical practice; (ii) different patterns compared to healthy subjects or severity are present; (iii) a relationship with validated functional measures is found; (iv) a response to rehabilitation congruent with validated measures is observable; and (v) the values assessed at baseline may predict rehabilitative outcomes.

## 2. Materials and Methods

This prospective trial (NCT: 02267798) was conducted at the Unit of Rehabilitation Medicine at the University Hospital of Ferrara, Italy. The local ethics committee of Ferrara University Hospital approved the study (number 101/12, on 27 September 2012). The research was carried out according to the Code of Ethics of the World Medical Association (Declaration of Helsinki), and written informed consent was obtained from all participants.

Forty patients with a first, single unilateral ischemic stroke within 8 weeks from the onset were proposed to participate. To be enrolled in the study, patients had to have upper limb motor impairment defined by an upper extremity score >11 and <55 on the Fugl–Meyer Assessment (FMA-UE). Patients were excluded if they presented medical conditions interfering with a safe completion of the protocol, impaired cognitive functioning, or severe upper limb pain.

Patients were then randomized according to a block randomization approach to receive hand functional electrical stimulation during robot-assisted therapy or conventional therapy during their hospital stay for a total of 6 weeks [23]. More details related to the study protocol are reported elsewhere [23].

For the present study, an additional sample of six age-matched healthy control subjects was also measured (aged 55 ± 11 years, 66% males).

### 2.1. Outcome Measures

Outcome measures were collected for all subjects at baseline and for patients at the end of treatment. For this subanalysis of the main trial, the following assessments were considered.

#### 2.1.1. Assessment of Cortical Oxygenation by Near-Infrared Spectroscopy

An analysis model was developed to quantify the variations in oxygenation that occurred during the motor task of reaching and grasping for the hemiparetic arm, as proposed by Kato et al. [10].

Each patient was equipped with a NIRS system (NIRScout, NIRx Medical Technologies LLC, Glen Head, NY, USA) composed of 16 sources and 16 detectors emitting two wavelengths of near-infrared light (760 and 850 nm). Hemodynamic signals were recorded at a sampling rate of 3.46 Hz. A standard cap was placed over each participant’s scalp, and sources and detectors were positioned on the measuring cap according to the 10–20 international system. The spatial distribution of the optodes on the cap was chosen to create channels (i.e., source-detector pairs) with standard interoptode distances of approximately 3 cm. Optodes were placed over both hemispheres, resulting in 48 channels covering the regions of the primary motor and sensorimotor cortices.

After collection, data were analyzed using NIRSlab software (v. 2017.6, NIRx Medical Technologies LLC, Glen Head, NY, USA). After checking the data quality by removing discontinuities, spikes, and movement artifacts by proper tools, a bandpass filter was applied. Channels with a signal-to-noise ratio that was too high or with a specific gain >7 were removed. Patients who presented more than eight low-quality channels were excluded from the analysis. Then, the optical signals of each channel were converted to oxygenated (oxy-Hb) and deoxygenated hemoglobin concentration changes by using the modified Beer–Lambert law [24]. For the present study, the change in Oxy-Hb was considered, being a signal correlated with the change in regional cerebral blood flow reflecting the variations in neural activity [25].

Then, each patient, while sitting on a standard chair with both arms laying on a fixed table, was instructed to perform the motor task of reaching and grasping with the hemiparetic arm. The task, which lasted for 15 s spaced out by 45 s of rest, was repeated six times. For quantification of cortical oxygenation, we calculated the area under the curve (AUC) of the Oxy-Hb trace by summing all the values during the 15 s reaching and grasping task for each of the 48 channels (Total-AUC, TOT-_AUC_). The mean value for the six repetitions of the task was then calculated and employed for data analyses. The AUCs of both hemispheres (24 channels each) were also calculated, defined as AFF-_AUC_ for the affected hemisphere and UN-_AUC_ for the contralateral hemisphere.

**Figure 1:**
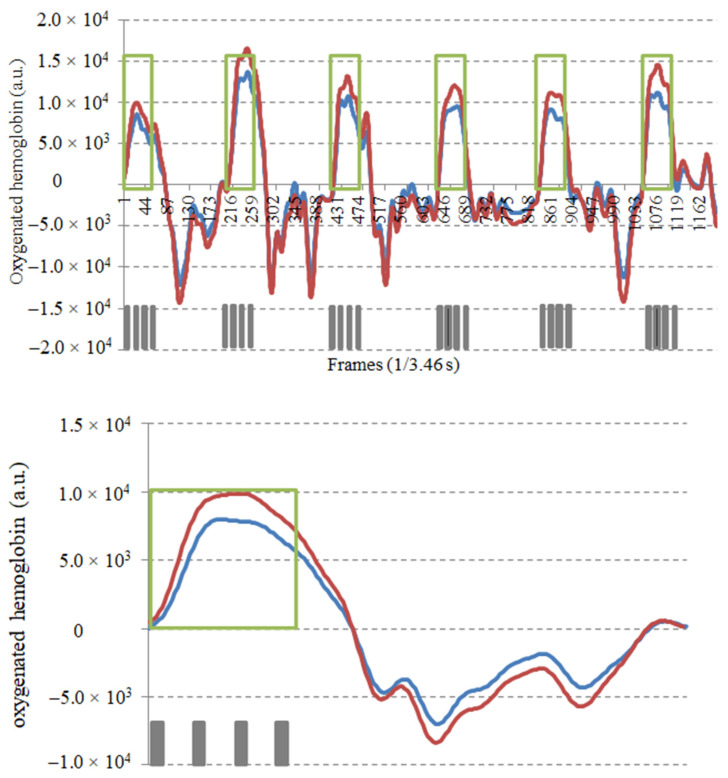
Method of calculation of cortical oxygenation. TOP: mean track of the 24 channels for each hemisphere during the six reaching and grasping tasks (represented in green; red: affected hemisphere; blue: unaffected hemisphere). BOTTOM: Mean traces of the six reaching and grasping cycles. In grey are represented the number of reaching and grasping repetitions performed by the patient. Cortical oxygenation (AUC) was calculated as the area under the curve for both hemispheres. The CMC was obtained by dividing each AUC for the number of repetitions.

In addition, the total number of reaching and grasping cycles was collected. The calculated AUC was then divided by the number of repetitions performed by the patient for every 15 s to obtain the measure of the cortical metabolic cost (CMC) of a single reaching and grasping movement. The CMC was obtained for the total primary and sensory motor cortices (TOT-_CMC_) and for both affected and unaffected hemispheres (AFF-_CMC_ and UN-_CMC_, respectively) (Figure 1).

#### 2.1.2. Upper Limb Motor Impairment

For the evaluation of upper arm motor recovery in stroke patients only, the Fugl–Meyer Motor Assessment score was considered in this analysis. The FMA-UE is a scale with a score that ranges from 0 to 66 and is widely used in rehabilitation trials for stroke patients [26].

### 2.2. Statistical Analysis

Data distribution was assessed using the Shapiro–Wilk test. The baseline characteristics of the patients are reported as the mean ± standard deviation or median (interquartile range) according to the data distribution. Interindividual differences were assessed through independent samples t tests or Mann–Whitney tests. Within-patient variations after rehabilitation were assessed using a paired samples t-test or Wilcoxon signed-rank test, as appropriate. Correlations between different outcome measures were assessed using Spearman’s rho.

A *p* value < 0.05 was considered statistically significant. Data analysis was performed with MedCalc Statistical Software v. 20.014 (MedCalc, Ostend, Belgium).

## 3. Results

Of the 40 patients enrolled in the trial [23], 2 were excluded because they were not willing to undergo NIRS measurements. Among the 38 patients enrolled, the test was safely conducted in all patients, lasting a mean time of 20 min, including setup and motor tasks. The analysis, requiring approximately 60 min for data decoding of each test, was performed in 23 patients. Fifteen patients were excluded because they presented at least eight channels with a high gain due to hair that interfered with the capability of the NIRS optodes to penetrate enough in the skull to have a secure reading of the brain cortex.

The baseline characteristics of the patients included in this analysis are reported in Table 1.

For the included patients, both AUC and CMC were successfully analyzed.

**Figure 2:**
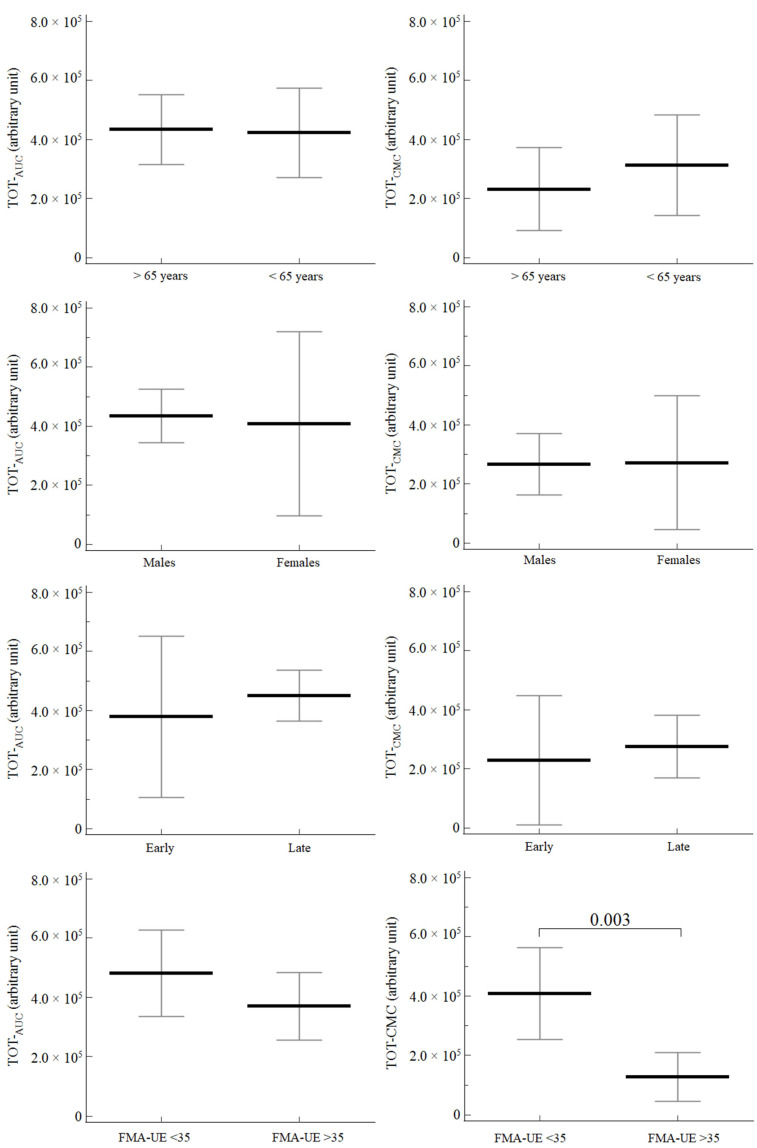
Baseline comparison of values of TOT-_AUC_ and TOT-_CMC_ according to age, sex, distance from stroke onset (early or late) and FMA-UE score. Data are reported as mean (95% confidence interval).

At baseline, TOT-_AUC_, AFF-_AUC_ and UN-_AUC_ did not differ by sex, age class (> or <65 years), baseline upper arm function (FMA-UE cutoff set at 35) or distance from stroke to rehabilitation (> or <30 days) (Figure 2).

No differences were noted for CMC considering age, sex and distance from stroke onset. Significantly higher values were noted in relation to the baseline value of FMA-UE for the more impaired subgroup for TOT-_CMC_ (*p* = 0.003), AFF-_CMC_ (*p* = 0.013) and UN-_CMC_ (*p* = 0.018) (Figure 2).

### 3.1. Comparison with Healthy Subjects

The AUC and CMC values for stroke survivors were significantly higher than those obtained by a group of age- and sex-matched control subjects, considering both total area and single hemispheres. The values are reported in Table 2.

### 3.2. Correlation between Cortical Oxygenation and Residual Upper Arm Motor Function

**Figure 3:**
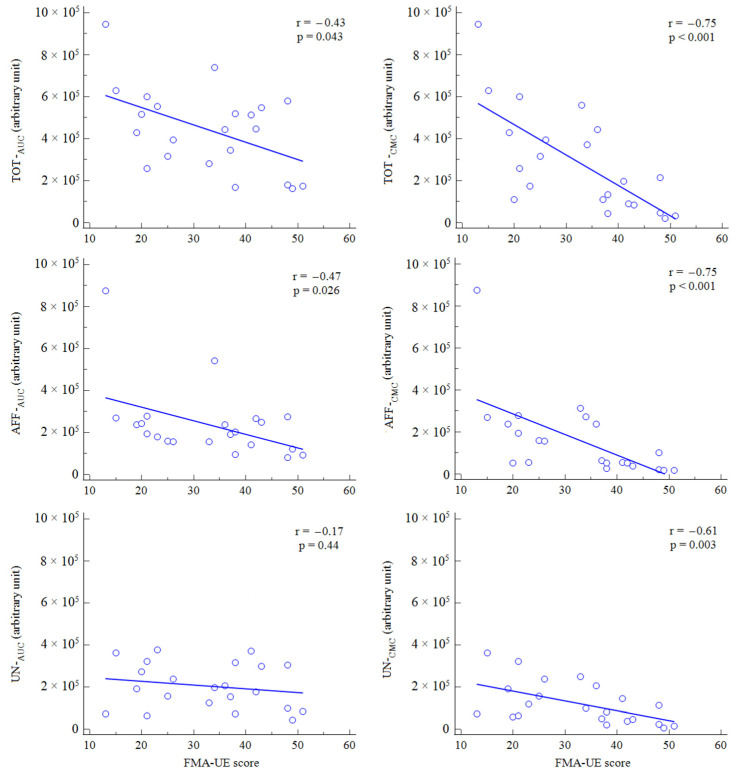
Rank correlation between AUC (**left**) or CMC (**right**) and FMA-UE score at baseline.

Cortical oxygenation was correlated with the FMA-UE score for both TOT-_AUC_ and AFF-_AUC_ but not for UN-_AUC_. Upper arm function was also significantly correlated with TOT-_CMC_, AFF-_CMC_ and UN-_CMC_. Data are reported in Figure 3.

### 3.3. Variations in Cortical Oxygenation following Rehabilitation

After rehabilitation, a significant increase in AUC was observed for both the AFF-_AUC_ (t = 1.80 *p* = 0.048) and UN-_AUC_ hemispheres (t = 1.90 *p* = 0.027).

For CMC, a decreasing trend was observed for AFF-_CMC_ (t = −1.35 *p* = 0.19), and a stable trend was observed for UN-_CMC_ (t = 0.03 *p* = 0.98).

Variations in TOT-_CMC_ were independent of sex (*p* = 0.81), age class (*p* = 0.77), rehabilitation treatment (*p* = 0.65) and baseline FMA-UE [27] (*p* = 0.78). No differences were noted for AFF-_CMC_ and UN-_CMC_ and also for TOT-_AUC_. 

**Figure 4:**
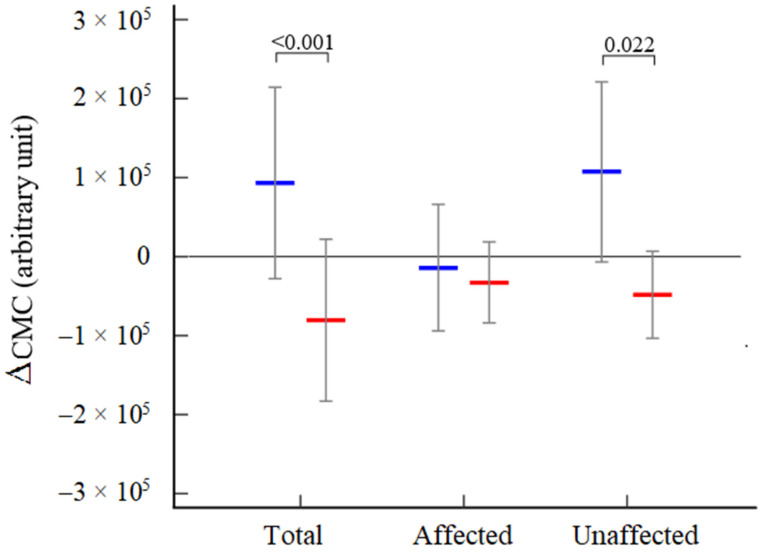
Variations in CMC for the total and both affected and unaffected hemispheres according to access to rehabilitation. Legend: Early (<30 days) blue line; Late (>30 days) red line.

As a final observation, variations after rehabilitation of Tot-_CMC_ and UN-_CMC_, but not AFF-_CMC_, were significantly different according to early or late access to rehabilitation (cutoff < 30 days) Figure 4.

### 3.4. Relationship between Cortical Oxygenation and Hemiparetic Upper Arm Motor Improvement 

**Figure 5:**
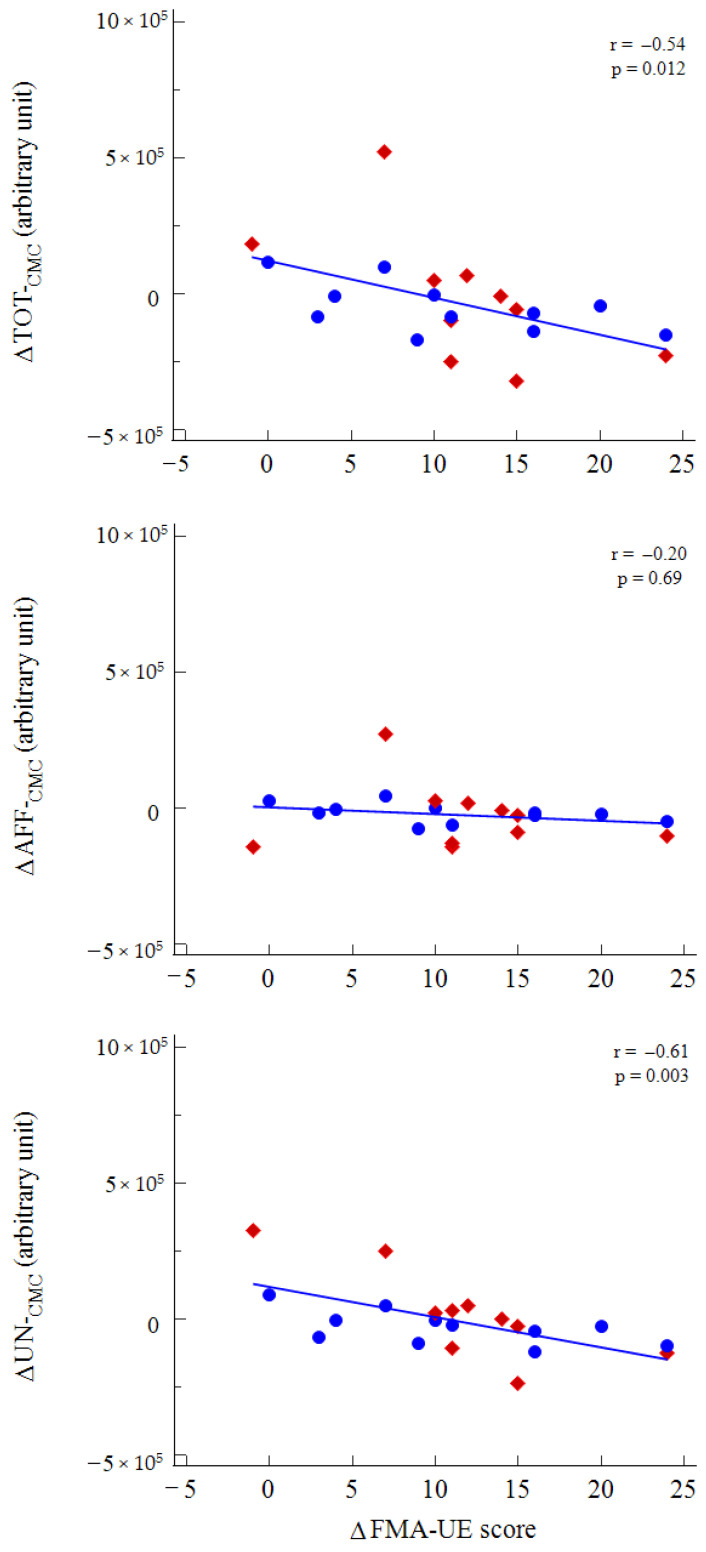
Rank correlation between variations of CMC and variations of FMA-UE score following rehabilitation. Legend: red diamonds, conventional therapy; blue dots, robotic rehabilitation.

Variations in the AUC and CMC of the total area and of the unaffected hemisphere were significantly inversely correlated with variations in both FMA-UE, with more favorable variations in upper arm motor function related to a decrease in CMC and total AUC Figure 5. 

### 3.5. Response of the CMC for Each Hemisphere following Rehabilitation

The contribution for the task movement of the two hemispheres was calculated by dividing the AFF-_CMC_ or the UN-_CMC_ for the TOT-_CMC_ to obtain the percent contribution to the movement.

**Figure 6:**
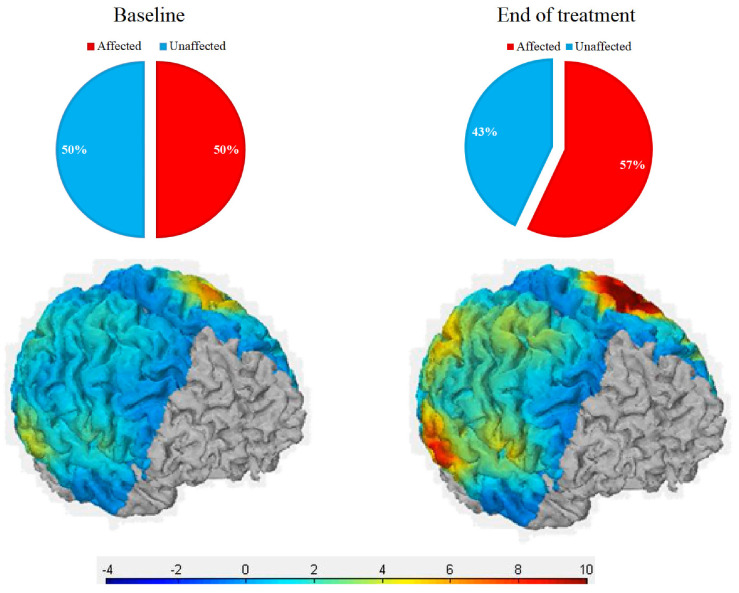
Representation of mean cortical oxygenation during the motor task for the affected (**front**) and unaffected (**back**) hemispheres. Legend: scale of cortical oxygenation to be multiplied by 10 E5 units.

The contribution of the affected hemisphere to the motor task progressively increased from a baseline value of 50% to a 57% total at the end of treatment (*p* = 0.016), with a concomitant decrease in the contribution of unaffected hemisphere (from 50% to 43%). A sample of the cortical oxygenation of a single subject is reported in Figure 6.

### 3.6. Baseline Cortical Oxygenation and Hemiparetic Upper Arm Motor Improvement

No significant relations were observed when comparing baseline TOT-_CMC_, AFF-_CMC_ and UN-_CMC_ and variations in FMA-UE scores.

Since a significant difference was noted in the response of cortical oxygenation in early and late stroke, we normalized the baseline cortical oxygenation by dividing each NIRS parameter by the number of days elapsed since stroke.

A significant inverse correlation was found for baseline TOT-_CMC/days_ and AFF-_CMC/days_ with variations in FMA-UE at the end of rehabilitation (r = −0.49; *p* = 0.026 and r = −0.50; *p* = 0.022, respectively).

**Figure 7:**
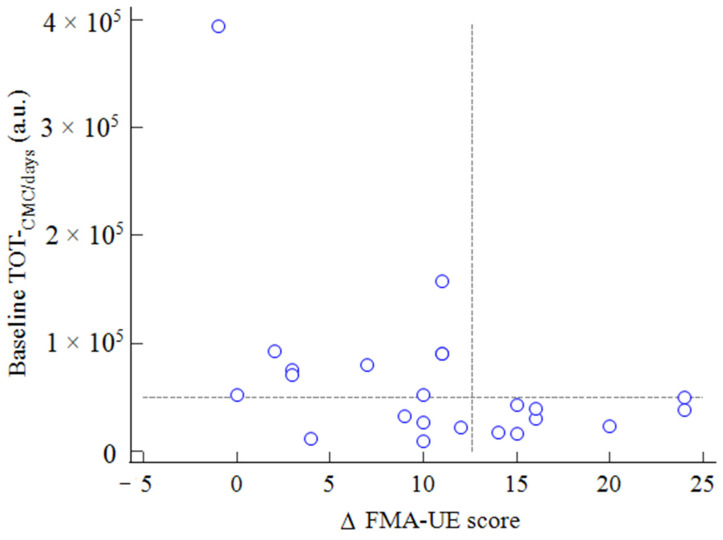
Rank correlation between baseline TOT-_CMC/days_ and variations in the FMA-UE score after rehabilitation. The vertical reference line corresponds to the minimal clinically important difference for FMA-UE.

Interestingly, none of the patients with a baseline TOT-_CMC/days_ > 5000 a.u. reached the minimal clinically important difference (MCID) for FMA-UE (values > 12.4 [28]) Figure 7. 

## 4. Discussion

A functional test combined with NIRS monitoring in patients with subacute stroke and arm paresis allowed us to measure the degree of cortical oxygenation during a standardized upper-arm motor task to transform it into a parameter of metabolic cost. The suitability of this parameter as a potential biomarker of brain plasticity in stroke recovery was also tested.

The protocol used in the study, inspired by the study of Kato et al. [10], was feasible in the execution phase and without disturbance for the patients. This NIRS-derived technological biomarker candidate was tested from different perspectives; in the first instance it differentiated healthy persons from diseased persons. Compared with values collected in healthy persons during the execution of the same task, a hyperactivation was observed in poststroke survivors, who showed TOT-_AUC_ values 3- to 10-fold higher to perform significantly less work. Purposely, the analysis was also normalized to the maximum number of actions performed during the duration of the task to obtain a parameter of cortical metabolic cost required to sustain a single movement with the affected arm. The CMC highlighted the difference between the two groups with a metabolic cost per single action even 50 times higher in diseased subjects. Among these patients, both AUC and CMC were inversely correlated with their FMA-UE score, but interestingly and not surprisingly, higher correlations were found for CMC both for total and for each hemisphere, including the unaffected hemisphere. This fact allows us to observe that approximately 40% of total CMC at baseline was supported by the affected hemisphere and a 60% by the unaffected hemisphere in patients with very low FMA-UE scores (<20) with a narrower range of values for people with less marked disability (>20). After rehabilitation, a generalized increase in TOT-_AUC_ was observed, but not for TOT-_CMC_ in view of the increased number of repetitions performed, with the CMC of the affected hemisphere which showed a trend to decrease and the nonaffected hemisphere which was stable.

Focusing therefore on the CMC parameter, it was observed that after rehabilitation, and simultaneously at a greater distance from the event, its values changed significantly, with a trend congruent with the FMA-UE and with a selective response. In particular, a greater decline in UN-_CMC_, which accounts for the variation in the TOT-_CMC_, was associated with a greater recovery as assessed by the changes in FMA-UE.

However, the protocol simply enabled the quantification of the metabolic engagement of each hemisphere to support upper limb movement in the poststroke phase. The present study is therefore limited to highlighting their floating contribution following the combined plastic stimuli from rehabilitation and time. Otherwise, the aspects of disturbance and recovery of interhemispheric balance, which has been well addressed by various authors, also in the implications related to the recovery of function, were not examined [10,15,21]. As previously reported, after stroke, an imbalance between hemispheres has been found, with increased activity of the unaffected hemisphere and decreased activity of the affected hemisphere [10,21]. During arm movements, an activation of both hemispheres was detected in our sample, revealing how the unaffected hemisphere played a significant role in movement execution early after stroke. Our findings are similar to those of other fNIRS studies performed on stroke patients [10,21].

A further interesting aspect is the possible predictive potential of CMC in terms of functional recovery. From this perspective, we had to consider the time-related spontaneous neurobiological recovery related to motor function recovery [29,30]. At baseline, CMC was slightly higher in older people and significantly higher in the early enrolled patients. Additionally, the CMC variations following the rehabilitative phase were significantly greater for TOT-_CMC_ and unaffected hemisphere in the subgroup at early enrollment than in the subgroup at late enrollment. For this reason, the baseline CMC value was normalized to the number of days elapsed since the event to explore a potential relationship of this parameter with the functional outcomes at discharge. Interestingly, only patients with values below 5000 a.u./day at the first measurement showed an FMA-UE improvement equal to or superior to the MCID [28] at the end of the rehabilitative phase, independently of being enrolled early or late. This information, when confirmed, might be of clinical use in the rehabilitative setting to identify a group of patients with potentially more favorable outcomes in the recovery of upper limb function.

The strengths of the study are related to the identification of a potential clinical biomarker of functional recovery of the hemiparetic arm, to the fact that it was carried out on a relatively large number of subjects considering the available literature [15], to the longitudinal measurements performed [9] and to the validation tested in several respects.

However, there are numerous limitations to the study, partly related to the NIRS technique. In particular, the test was performed in a sample of patients only, with a high number of excluded patients. This particular aspect has now been corrected through updated caps and optode holders. The optode positioning might have been slightly different between each subject, even though the same skilled operator performed all the measurements. Moreover, short-channel measurements to correct the data for possible confounders were not performed. The analysis was focused on the performance of the hemiparetic arm only. The study also did not discuss the possible different responses in terms of CMC between the two rehabilitative treatments, which were not different in terms of effectiveness in the trial [23], and which presented similar variations in TOT-_CMC_ values after rehabilitation treatments (data not shown). 

Finally, a significant limitation may be related to the lack of electrophysiological studies to directly measure the poststroke cortical activity. Despite the neurovascular coupling phenomenon and the correlation between neuronal activity and oxygenation previously reported, the fact that neurovascular coupling is disturbed in energetically unfavorable conditions like poststroke, should be considered [31,32]. To this end, the scientific literature has defined fNIRS as a reliable measure of cortical activity also in stroke survivors [15,33,34], when proper methods of analyses, as those used in this study, are performed [33]. In addition, recent studies using simultaneous electroencephalography (EEG)-NIRS measurement have shown that fluctuations in EEG and NIRS signals are correlated [22,34,35].

## 5. Conclusions

In conclusion, NIRS recording during a motor task involving the upper limb was feasible and allowed us to identify a potential biomarker of functional recovery after stroke based on the cortical metabolic cost of actions. The CMC parameter was significantly altered in the poststroke patients, with values correlated with those derived from validated measures, responsive to time/treatments, selective in hemispheric participation and predictive of clinical recovery following rehabilitation.

If these preliminary data were confirmed, this biomarker may add information to routine evaluations to study hemispheric participation in the poststroke phase, to draw trajectories of recovery as a function of time and treatment and to identify the possible responders to rehabilitation. Larger validation studies are therefore necessary.

## Figures and Tables

**Table 1:** Baseline characteristics of the included patients.

	Stroke Patients Analyzed (*n* = 23)
Age, years	68 (58–73)
Males, *n* (%)	14 (61)
Time since stroke, days	46 ± 20
Left affected hemisphere, *n* (%)	16 (70)
Sensory impairment, *n* (%)	6 (25)

**Table 2:** Comparison between stroke patients and healthy subjects for AUC and CMC.

	Stroke (*n* = 23)	Healthy (*n* = 6)	*p*
TOT-_AUC_	4.3 ± 2.1	0.6 ± 0.1	<0.001
AFF-_AUC_	2.3 ± 1.7	0.5 ± 0.1	<0.001
UN-_AUC_	1.9 ± 1.1	0.1 ± 0.03	<0.001
TOT-_CMC_	2.7 ± 2.4	0.5 ± 0.1	<0.001
AF-_CMC_	1.6 ± 1.8	0.4 ± 0.01	<0.001
UN-_CMC_	1.2 ± 1.0	0.1 ± 0.04	<0.001

Legend: in healthy subjects, the affected hemisphere was considered the nondominant hemisphere. Values reported are divided by 10^5^ arbitrary units.

## Data Availability

Data are available upon request to Nicola Lamberti (nicola.lamberti@unife.it).
